# Proteasome Activity Influences UV-Mediated Subnuclear Localization Changes of NPM

**DOI:** 10.1371/journal.pone.0059096

**Published:** 2013-03-12

**Authors:** Henna M. Moore, Baoyan Bai, Olli Matilainen, Laureen Colis, Karita Peltonen, Marikki Laiho

**Affiliations:** 1 Molecular Cancer Biology Program, University of Helsinki, Helsinki, Finland; 2 Sidney Kimmel Comprehensive Cancer Center and Department of Radiation Oncology and Molecular Radiation Sciences, Johns Hopkins University School of Medicine, Baltimore, Maryland, United States of America; UMCG, The Netherlands

## Abstract

UV damage activates cellular stress signaling pathways, causes DNA helix distortions and inhibits transcription by RNA polymerases I and II. In particular, the nucleolus, which is the site of RNA polymerase I transcription and ribosome biogenesis, disintegrates following UV damage. The disintegration is characterized by reorganization of the subnucleolar structures and change of localization of many nucleolar proteins. Here we have queried the basis of localization change of nucleophosmin (NPM), a nucleolar granular component protein, which is increasingly detected in the nucleoplasm following UV radiation. Using photobleaching experiments of NPM-fluorescent fusion protein in live human cells we show that NPM mobility increases after UV damage. However, we show that the increase in NPM nucleoplasmic abundance after UV is independent of UV-activated cellular stress and DNA damage signaling pathways. Unexpectedly, we find that proteasome activity affects NPM redistribution. NPM nucleolar expression was maintained when the UV-treated cells were exposed to proteasome inhibitors or when the expression of proteasome subunits was inhibited using RNAi. However, there was no evidence of increased NPM turnover in the UV damaged cells, or that ubiquitin or ubiquitin recycling affected NPM localization. These findings suggest that proteasome activity couples to nucleolar protein localizations in UV damage stress.

## Introduction

The nucleolus is a membraneless nuclear organelle that governs ribosome biogenesis. It is physically formed around hundreds of ribosomal gene repeats. In the nucleolus, RNA polymerase I (Pol I) transcribes ribosomal (r) DNA into rRNA [1], [2]. The nucleolus is composed of substructures, which correspond to the vectorial movement and processing of the maturing rRNA transcripts. In higher eukaryotes, transcription of the 47S rRNA precursor is initiated at the border of fibrillar centers (FC) and dense fibrillar centers (DFC). The 47S transcript is then cleaved to 28S, 18S and 5.8S rRNAs [3], [4]. The transcripts are further modified in the DFC, and assembled in the granular component (GC) together with ribosomal proteins and 5S RNA into ribosomal subunits, which are then transported to cytoplasm where fully active ribosomes are formed [3]. Since ribosomes are prerequisite for all cellular protein production their amount is rate limiting in cell proliferation. 50% or more of total cellular transcription of rapidly proliferating cells results from rRNA transcription. Therefore, ribosome biogenesis and the synthesis of rRNA is strictly controlled [5], [6].

The nucleolus harbors a substantial number of distinct proteins requisite for the rRNA biogenesis. More than 4500 proteins have been identified in the nucleolus [7], several of which are highly dynamic within their subcellular localization [8], [9]. Due to the divergent functions of the nucleolar proteome, the nucleolus has been proposed to participate in additional cellular processes. Nucleolar proteins have been reported to regulate tumor suppressor protein and oncogene activities, cell cycle, signal recognition particle assembly, to modify small RNAs, control aging and telomerase function, to regulate mitosis, cell growth and death, and to function as sensors for cellular stress [10]–[14]. In addition, many ribosomal proteins have extra-ribosomal functions that are disconnected of ribosome biogenesis [15], [16].

We have previously shown that a multifunctional and an abundant nucleolar protein nucleophosmin (NPM, B23) relocalizes from the nucleolus to the nucleoplasm following UV damage [17]. UV radiation is a major environmental carcinogen, which causes formation of DNA helix distorting adducts [18]. These form physical barriers that halt the transcription by RNA polymerases and evoke complex cellular stress responses [19]. To date, it is not known what controls the change in NPM localization after UV radiation. Consequent to UV-mediated NPM relocalization to the nucleoplasm it binds MDM2 and protects p53 from MDM2-mediated proteasomal degradation [17]. In addition, similar functions have been published for several ribosomal proteins in a process termed as nucleolar or ribosomal stress, where nucleolar disruption is followed by p53 stabilization [20], [21]. We have recently detailed, using quantitative proteomics and cellular imaging, the responses of hundreds of nucleolar proteins to DNA damage caused by UV and ionizing radiation [22]. We showed that the nucleolar expression of a marked number of proteins changes after UV, while the changes following ionizing radiation are less dynamic and involve only a subset of proteins. What directs these dynamic changes is unknown.

Protein degradation is an essential cellular process, in which excess and misfolded proteins are degraded. The major degradation pathway in eukaryotic cells is the ubiquitin-proteasome system, where ubiquitin is repeatedly added to targeted proteins by specific enzymes (E1, E2 and E3) in a strictly controlled manner [23]. Polyubiquitin chains formed by K48 and K11-linkages are recognized by the proteasome leading to degradation of polyubiquitinated proteins. Inhibition of proteasome function causes accumulation of polyubiquitinated proteins, which may lead to severe cellular stress and cell death. This feature is utilized in cancer therapy through the use of chemical proteasome inhibitors [24].

Recent evidence indicates a functional interplay between the nucleolus and proteasome function. Proteasome inhibitor treatment alters nucleolar morphology, inhibits nucleolar rRNA processing [25]–[27], and causes accumulation of ribosomal proteins in the nucleolus [28]. Ubiquitin has been detected in the nucleolus [25], also in the conjugated form [27], and is relevant in the clearance of nonfunctional ribosomes and rRNAs [29]. Several ribosomal proteins are conjugated by ubiquitin, or expressed as ubiquitin-fusion proteins [21], [30], [31]. 20S proteasome core has been detected in the nucleolus in certain conditions [27], [32], [33] although there are reports that contrast this result [34]. It has been suggested that the nucleolus directly controls the proteasomal degradation of certain proteins, like c-Myc and p53 [33], [35]. We have recently identified a nucleolus-associated RNA-protein aggregate, which forms following proteasome inhibition, and is alleviated by ectopic expression of ubiquitin suggesting that inhibition of ubiquitin recycling contributes to the nucleolar accumulation [27]. Finally, a nucleolar deubiquitinase USP36 regulates nucleolar activity by affecting nucleolar morphology and inhibiting rRNA transcription and processing [36]. The majority of functional links between the nucleolus and proteasome implicates association of the ubiquitin pathway in nucleolar control.

We investigate here the UV damage-activated processes that relate to the changes in localization of nucleolar proteins. In this context, we considered pathways relevant in UV- mediated intracellular stress signaling, DNA damage signaling and the proteasome activity. We show here that proteotoxic stress inhibits the UV radiation –activated relocation of NPM and other GC-proteins. Interestingly, it is independent of ubiquitin availability as demonstrated by genetic manipulation of several ubiquitin conjugating factors. Conversely, we show that genetic silencing of 20S proteasome core by RNAi leads to inhibition of UV damage –mediated NPM relocation, suggesting that the proteasome is essential for NPM localization change after UV stress.

## Results

### NPM nucleolar mobility is increased following UV damage

**Figure 1:**
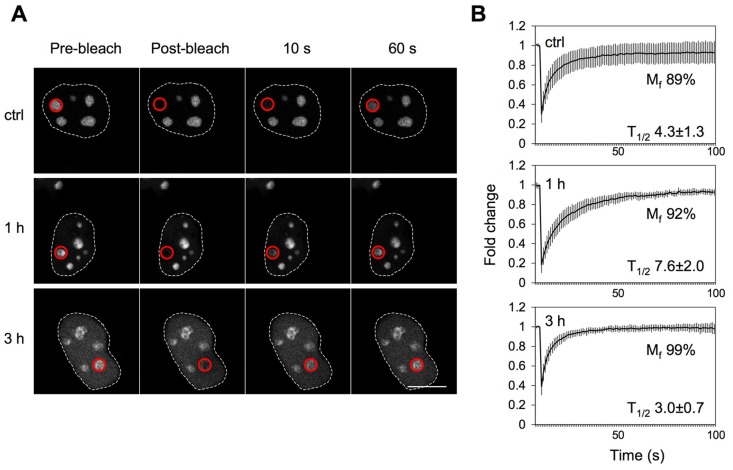
NPM nucleolar mobility is increased following UV radiation. *A* U2OS cells were transiently transfected with NPM-ECGFP and treated with UVC (35 J/m^2^) or left untreated (control). FRAP analysis was performed on a single nucleolus as indicated by ROI (*red* circle). Following photobleaching images were captured every 1 s for 100 s. Representative images are shown. Scale bar 10 µm. *B* Averages of normalized intensities, mobile fraction (M_f_) and recovery half times (T_1/2_) from at least two independent experiments for each treatment are shown. Error bars, SD. *N* ≥ 8 cells for each treatment.

NPM is highly mobile, and the mobility is further increased after inhibition of RNA Pol I by low doses of Actinomycin D [37]. We have shown a change in NPM localization from the nucleolus to the nucleoplasm following UV damage [17], and wanted hence to ascertain whether this is associated with a change in NPM mobility. We transiently transfected U2OS cells with NPM tagged with enhanced cyan green fluorescent protein (ECGFP) and used fluorescence recovery after photobleaching (FRAP) to record its intensity in nucleoli of untreated and UV-treated cells at different times after damage (Fig. 1*A*). The mobility of NPM-ECGFP was high already in untreated control cells as indicated by mobile fraction (M_f_) calculated from the intensity data (89%, Fig. 1*B*). Following UV damage, the mobility of NPM-ECGFP further increased to 92% and 99% at 1 and 3 hours after damage, respectively (Fig. 1*B*). We determined also protein recovery half times (T_1/2_), *i.e.* how fast NPM-ECGFP fluorescence recovers to half of the original level. UV damage affected recovery half times of NPM-ECGFP, changing from 4.3 seconds in control to 7.6 and 3.0 seconds at 1 and 3 hours after UV damage, respectively. Over time, NPM-ECGFP was increasingly detected in the nucleoplasm, and a similar FRAP-analysis indicated that the nucleoplasmic NPM-ECGFP was fully mobile (M_f_  = 100%, Fig. S1). These results indicate that after UV damage NPM mobility increases concomitant with a more prominent nucleoplasmic localization. The longer T_1/2_ observed 1 hour after UV damage may relate to transient NPM associations early after the UV damage and will need to be investigated in further in-depth imaging analyses.

### Proteotoxic stress inhibits UV damage–mediated NPM relocalization

**Figure 2:**
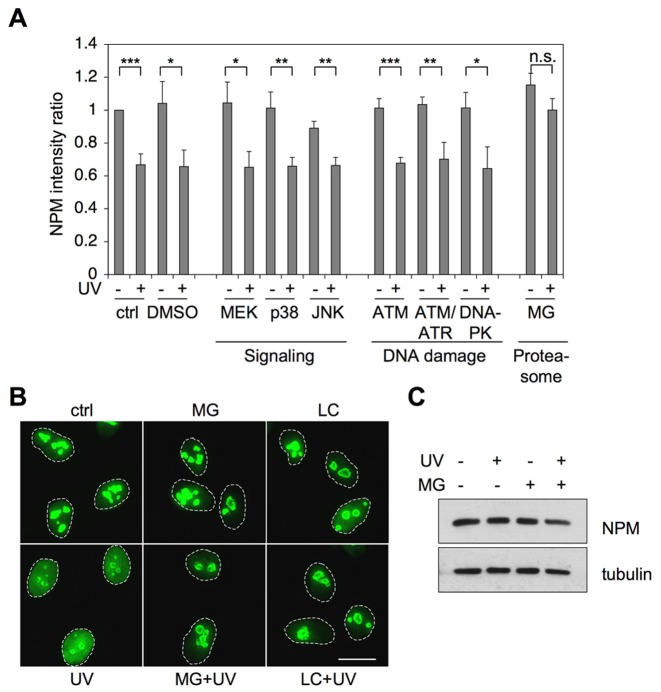
UV-activated NPM relocalization is prevented by treatment with proteasome inhibitor. *A* U2OS cells were treated with inhibitors targeting UV-activated cellular signaling (U0126 10 µM for MEK, SB203580 20 µM for p38 and SP600125 100 µM for JNK), DNA damage signaling (KU55933 10 µM for ATM, wortmannin 100 µM for ATM/ATR and NU7441 10 µM for DNA-PK) and proteasome (MG132 10 µM) or left untreated. One hour later the cells were exposed to UV radiation (35 J/m^2^) or left untreated. Cells were fixed after 3 hours and stained for NPM and UBF. Cells were imaged and intensities were quantified with Fiji-software using UBF as a nucleolar marker. The ratio of nucleolar and nucleoplasmic intensities was calculated from three independent experiments with two fields imaged per experiment. *P*-values were calculated using Student's T test, *P<0.05; **P<0.01; ***P<0.001. Error bars, SD. *N* ≥ 140 cells/analysis. *B* WS1 cells were treated with proteasome inhibitors MG132 (10 µM) or lactacystin (LC, 10 µM) for 1 hour prior to UV radiation (35 J/m^2^) or left untreated. The cells were fixed 6 hours later and stained for NPM. Scale bar 20 µm. *C* WS1 cells were treated with MG132 or left untreated. After 1 hour the cells were treated with UV radiation (35 J/m^2^) or left untreated. Cells were lysed 3 hours later into RIPA buffer. Equal amounts of total protein were separated by SDS-PAGE and immunoblotted for NPM. Tubulin was used as a loading control.

It is not known what causes NPM redistribution after UV damage. In order to query putative regulators of the process, we inhibited factors that function in signaling pathways activated by UV radiation and DNA damage. For this purpose we used specific inhibitors for MEK, p38, JNK, ATM, ATR/ATM, and DNA-PK pathways and pretreated cells with respective inhibitors for 1 hour before irradiation with UV. Since we have previously shown a link between proteasome activity and nucleolar function [27], we tested also a proteasome inhibitor in this setting. We fixed the cells after 3 hours and performed co-immunostaining for NPM and UBF. By using UBF as a nucleolar marker, we imaged and quantified the ratio of the nucleolar and nucleoplasmic NPM intensity (Fig. 2*A*). NPM localization ratio altered significantly in the control and UV-treated cells. However, none of the inhibitors that block UV-activated signaling pathways or DNA damage response pathways had any effect on the UV-mediated NPM translocation (Fig. 2*A* and Fig. S2). In contrast, proteasome inhibitor MG132 effectively inhibited NPM relocalization by UV damage (Fig. 2*A*).

We further confirmed the effect by using another specific proteasome inhibitor, lactacystin. WS1 cells were pre-treated with either MG132 or lactacystin for 1 hour followed by UV radiation. We fixed the cells after 6 hours, and performed immunostaining for NPM. Similarly to MG132, pretreatment with lactacystin inhibited NPM nucleoplasmic localization (Fig. 2*B*). In order to confirm that the effect was not selective for the NPM antibody used in the assay, we used U2OS cells stably expressing NPM-ECGFP and exposed them to UV in the presence or absence of MG132. MG132 inhibited NPM-ECGFP nucleoplasmic localization following UV similarly to the endogenous NPM (Fig. S3 *A*). In order to determine whether the effect was due to change in overall NPM protein level, we detected NPM expression by western blotting in WS1, U2OS and HeLa cells treated with MG132 and UV. There was no change in the total NPM protein level by UV or MG132 in any of the cell lines (Fig. 2*C*, Fig. S3 *B*). To further query whether UV damage changes NPM turnover, we assessed NPM stability in UV-treated cells by inhibiting *de novo* protein synthesis using cycloheximide. As shown in Figures S4*A* and *B*, there was no change in NPM half-life following UV treatment, nor did cycloheximide affect NPM localization (Fig. S4 *C*). Similarly, we addressed whether inhibition of RNA polymerase II transcription affects UV-dependent NPM localization using α-amanitin, and could not observe any change (Fig. S4 *D*). In conclusion, proteasome inhibitors MG132 and lactacystin inhibited the UV damage–mediated change in NPM localization without an apparent change in NPM expression.

### Proteasome inhibition decreases NPM mobility in UV-treated cells

**Figure 3:**
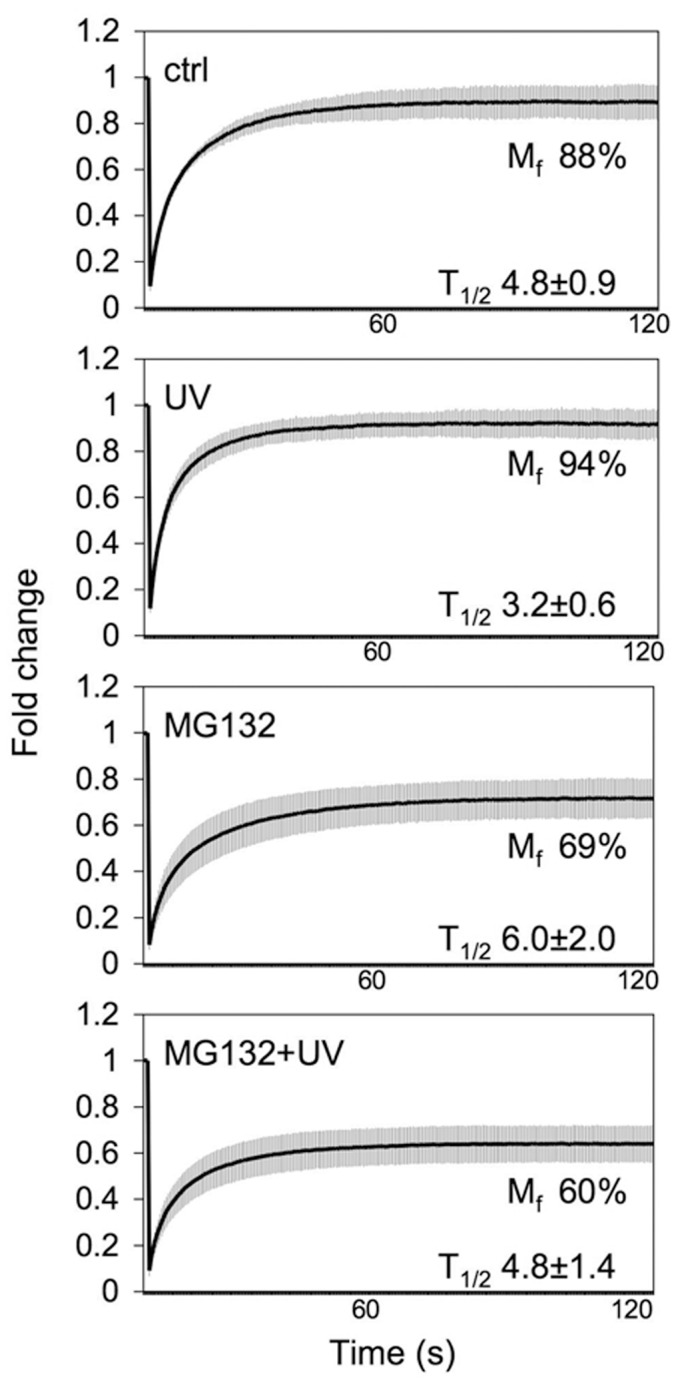
Nucleolar mobility of NPM is altered after proteasome inhibition and UV damage. U2OS cells stably expressing NPM-ECGFP were treated either with MG132 (10 µM) for 4 hours, UV (35 J/m^2^), pretreated with MG132 for 1 hour followed by UV treatment (35 J/m^2^) and incubation for 3 hours, or left untreated (control). Averages of normalized intensities, mobile fractions (M_f_) and recovery half-times (T_1/2_) from at least three independent experiments for each treatment are shown. Error bars, SD. *N = *5–8 cells for each treatment.

As shown in Figure 1, UV treatment increased the mobility of NPM-ECGFP. As proteasome inhibition has been shown to affect the mobility of certain nucleolar proteins, including NPM [25], [27], we wanted to test whether NPM mobility was affected by MG132 treatment in combination with UV damage. We performed FRAP–experiments on U2OS cells stably expressing NPM-ECGFP after treating the cells with UV, MG132 or their combination (Fig. 3). Whereas UV damage increased NPM-ECGFP mobility (M_f_ 94% as compared to control 88%), MG132 decreased the mobility (M_f_ 69%). Interestingly, in cells treated with both MG132 and UV, NPM-ECGFP mobility was further decreased (M_f_ 60%). Similar recovery half times were observed for control and UV-treated cells as in Fig. 1. The T_1/2_ in MG132-treated cells was slightly delayed as compared to control. However, in cells exposed to both MG132 and UV, the T_1/2_ was indistinguishable from control indicating that despite decreased mobility, the recovery half time was maintained (Fig. 3). This indicates that proteasome inhibition affects NPM mobility even in the context of UV damage.

### Effects of proteasome inhibition on nucleolar protein localization are not limited to NPM

**Figure 4:**
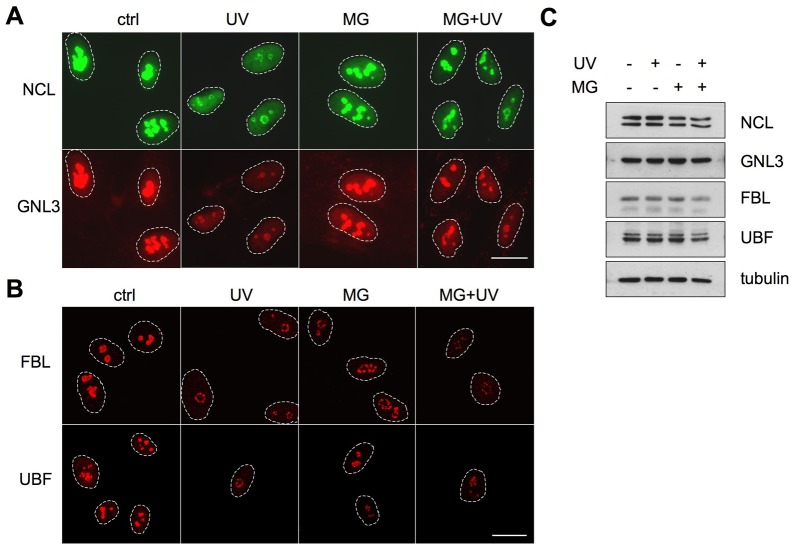
Nucleolar protein UV responses and proteasome inhibition are divergent and depend on the nucleolar subcompartment. WS1 cells were pretreated with MG132 followed by UV radiation (35 J/m^2^) as shown. Cells were fixed after 3 hours and stained for NCL and GNL3 (*A*), or FBL and UBF (*B*). Confocal images are shown for FBL and UBF (*B*). Scale bar 20 µm. *C* Western blotting analysis for the respective proteins. Equal amounts of total protein were separated by SDS-PAGE and immunoblotted for NCL, GNL3, FBL and UBF. Tubulin was used as a loading control.

Next we wanted to test whether proteasome inhibition affects the UV-mediated localization change of also other nucleolar proteins. We assayed for localization of nucleolar proteins with specific localizations in nucleolar substructures, FC, DFC and GC. We treated WS1 cells with MG132 and UV and immunostained the cells for GC-proteins nucleolin (NCL) and nucleostemin (GNL3). UV damage decreased nucleolar staining intensity of both NCL and GNL3, whereas pretreatment of the cells with MG132 inhibited both effects (Fig. 4*A*). DFC protein fibrillarin (FBL) and FC protein UBF did not display nucleoplasmic localization following UV (Fig. 4*B*). Rather, both form nucleolar necklaces around the nucleolus following UV [22] and transcriptional inhibition [38]. MG132-treatment, which alters the nucleolar substructures [27], did not inhibit DFC and FC protein reorganization following UV (Fig. 4*B*). As determined by western blotting there was no change in the expression of NCL, GNL3, FBL or UBF (Fig. 4*C*).

### rRNA biogenesis is inhibited at different stages by UV and proteasome inhibition

**Figure 5:**
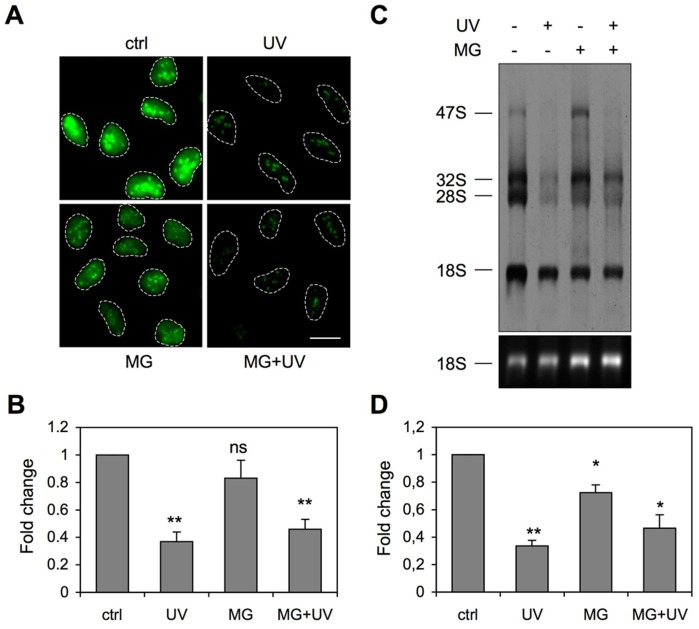
rRNA transcription and processing are inhibited after proteasome inhibition and UV radiation. *A* U2OS cells were pretreated with MG132 followed by UV radiation (35 J/m^2^) as shown. Cells were incubated for 3 hours and labeled with 1 mM EU for the last hour. Cells were fixed and EU labeling was detected by azide-containing dye. Scale bar 20 µm. *B* EU nuclear signal was quantified from two independent experiments. *P*-values were calculated using Student's T test, *P<0.05; **P<0.01; ***P<0.001. Error bars, SD. *N* = 51–70 cells for each analysis. *C* A375 cells were pretreated with MG132 followed by UV radiation (35 J/m^2^) as shown and incubated for 3 hours. Cells were labeled with ^3^H-uridine for the last 1 hour, and RNA was extracted. Equal amounts of RNA were separated by 1% agarose-formaldehyde gel and transferred onto nylon filter. Representative autoradiogram is shown and rRNA forms are indicated on the left. *D* ^3^H-uridine labeling was quantified by Fiji/ImageJ-software from two independent experiments. *P*-values were calculated by Student's T test, *P<0.05; **P<0.01; ***P<0.001. Error bars, SD.

UV radiation represses rRNA transcription [22], [39], [40], whereas MG132 inhibits late rRNA processing, but not rRNA synthesis [25]–[27]. We hence wanted to assess whether MG132 treatment impacts UV damage-mediated inhibition of rRNA transcription. First, we treated cells with UV in the presence or absence of MG132 alone and labeled the cells with ethynyl uridine (EU) for the last hour of incubation. Incorporation of EU was detected with azide-containing dye. UV radiation reduced the EU incorporation significantly, whereas MG132-treatment alone had only a minor, non-significant effect (Fig. 5*A and B*). MG132 had no effect on the UV-mediated repression of EU incorporation (Fig. 5*A and B*). To assess the synthesis and processing of the 47S rRNA to the mature 18S and 28S rRNAs, we used metabolic labeling of nascent rRNA with ^3^H-uridine. Cells were treated with MG132 and UV followed by incubation with ^3^H-uridine. RNA was extracted, separated in agarose gel and autoradiograms were obtained. UV radiation fully inhibited the synthesis of the pre-rRNA 47S transcript and decreased the levels of the 32S processed form and 28S mature rRNA (Fig. 5*C*). However, 18S rRNA was still detectable. MG132-treatment alone did not affect the 47S or 32S transcript synthesis indicating that the rRNA transcription or early processing *per se* was not affected (Fig. 5*C*). Expression of the 28S mature form was reduced suggesting inhibition of late processing. The quantified intensity of all rRNAs was lower in MG132-treated cells than in control (Fig. 5*D*). These results are in concordance with the earlier published results of MG132 as a processing inhibitor [26]. Finally, MG132-treatment did not rescue the UV-damage caused repression of rRNA synthesis as evident by the loss of the 47S transcript (Fig. 5*C*). These data show that proteasome inhibition and UV damage cause defects in rRNA biogenesis at different steps, and that proteasome inhibition does not compensate for the UV-mediated inhibition of rRNA synthesis.

### Ubiquitin recycling does not affect NPM response to UV and proteotoxic stress

**Figure 6:**
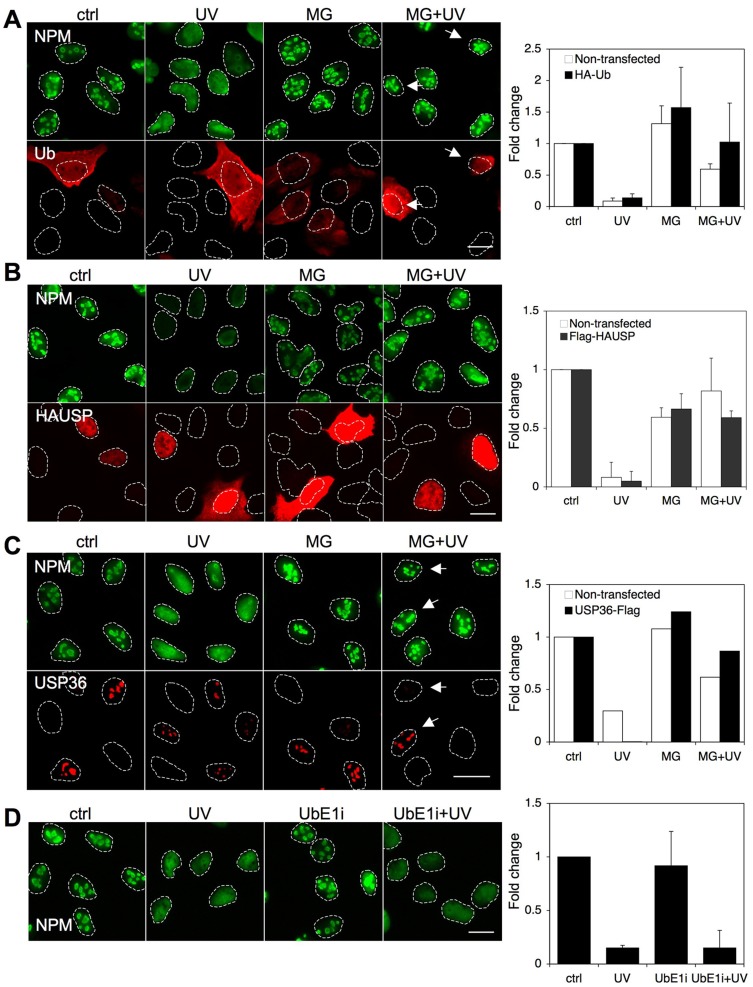
Ubiquitin recycling does not contribute to inhibition of NPM relocalization following UV radiation. U2OS cells were transfected with HA-tagged ubiquitin (*A*) or FLAG-tagged HAUSP (*B*). After 24 hours the cells were pretreated with MG132 followed by UV (35 J/m^2^) as shown and the cells were incubated for 6 hours. Cells were fixed and the expressed proteins were detected using HA- (*A*) or FLAG (*B*) -antibodies and co-stained for NPM. Nucleolar areas were quantified from three independent experiments. *C* U2OS cells stably expressing USP36-Flag were pretreated with MG132 followed by UV (35 J/m^2^) as shown and the cells were incubated for 3 hours. Cells were fixed and USP36 was detected using FLAG-antibody and cells were co-stained for NPM. Nucleolar areas were quantified. *D* U2OS cells were treated with UbE1 inhibitor (10 µM) or left untreated. After 24 hours the cells were exposed to UV (35 J/m^2^) and incubated for 3 hours. Cells were fixed and stained for NPM. Nucleolar areas were quantified from two independent experiments. Scale bars 20 µm.

Inhibition of the proteasome has two main effects on the cells. Due to inhibition of the catalytic activity of the proteasome, it leads to accumulation of polyubiquitinated proteins. Secondly, it leads to depletion of free ubiquitin normally released during processing of the polyubiquitinated proteins through the proteasome. Consequently, the lack of ubiquitin would also affect other processes, such as monoubiquitination, where the monoubiquitin tags serve as signals for protein localization or other specified functions. We have recently shown that ubiquitin availability is important in nucleolar function upon proteasome inhibition [27]. We therefore considered that ubiquitin tags might be relevant in the UV-mediated translocation of nucleolar proteins and become rate-limiting when cells were exposed to MG132 treatment. To assess this we overexpressed HA-tagged ubiquitin in U2OS cells and treated the cells with UV, MG132 or their combination. We fixed the cells and stained them for NPM and HA-ubiquitin. We imaged and quantified NPM nucleolar area in HA-tagged ubiquitin negative and positive cells separately. Overexpression of ubiquitin did not markedly affect the nucleolar retention of NPM in UV-treated cells by MG132 (Fig. 6*A*).

We then considered the possibility that ubiquitin tags themselves, present on the nucleolar proteins, would cause the retention of NPM in the nucleolus. Previously we showed that overexpression of HAUSP (herpesvirus-associated ubiquitin-specific protease, USP7) deubiquitinase counteracts nucleolar aggregate formation [27]. Hence we tested whether HAUSP affects NPM localization. We overexpressed Flag-tagged HAUSP in U2OS cells and determined NPM localization in UV and MG132-treated cells. Cells were stained for NPM and Flag-HAUSP. Quantification of NPM nucleolar area both in HAUSP negative and positive cells indicated that overexpression of Flag-HAUSP had no effect on NPM localization by any of the treatments (Fig. 6*B*). We also tested whether a nucleolar deubiquitinase USP36, which deubiquitinates NPM [41], affects the MG132-caused NPM nucleolar retention in the UV-treated cells. We stably expressed Flag-tagged USP36 in U2OS cells and treated the cells with UV radiation, MG132 or their combination. We fixed the cells and stained them for NPM and Flag-USP36. Quantified analysis of NPM indicated that expression of Flag-USP36 had no effect on NPM localization by any of the treatments (Fig. 6*C*).

MDM2, an E3 ligase for p53 has been suggested to be a potential regulator for GTP-depletion –induced nucleostemin redistribution [42], although this hypothesis has recently been challenged [43]. We therefore tested whether Nutlin-3, an inhibitor of MDM2 activity affects NPM localization. We treated U2OS cells with Nutlin-3, UV or their combination. Nutlin-3 had no effect on NPM localization, either alone or in UV–treated cells (Fig. S5).

We then tested whether ubiquitin conjugation affects NPM localization, and used a ubiquitin E1-ligase inhibitor [44] for this purpose. We pre-treated cells with UbE1-inhibitor for 24 hours followed by treatment of the cells with or without UV. We confirmed the activity of UbE1-inhibitor separately as detected by increased expression of p53 (Fig. S6). We fixed the cells after 3 hours, stained them for NPM, and imaged and quantified NPM nucleolar area. Treatment with UbE1-inhibitor had no effect on the UV-mediated NPM localization, suggesting that ubiquitin conjugation was not an essential mediator of NPM localization (Fig. 6*D*). In conclusion, manipulation of ubiquitin recycling by several different ways did not affect NPM translocation by UV damage.

### Inhibition of proteasome expression prevents NPM localization change

**Figure 7:**
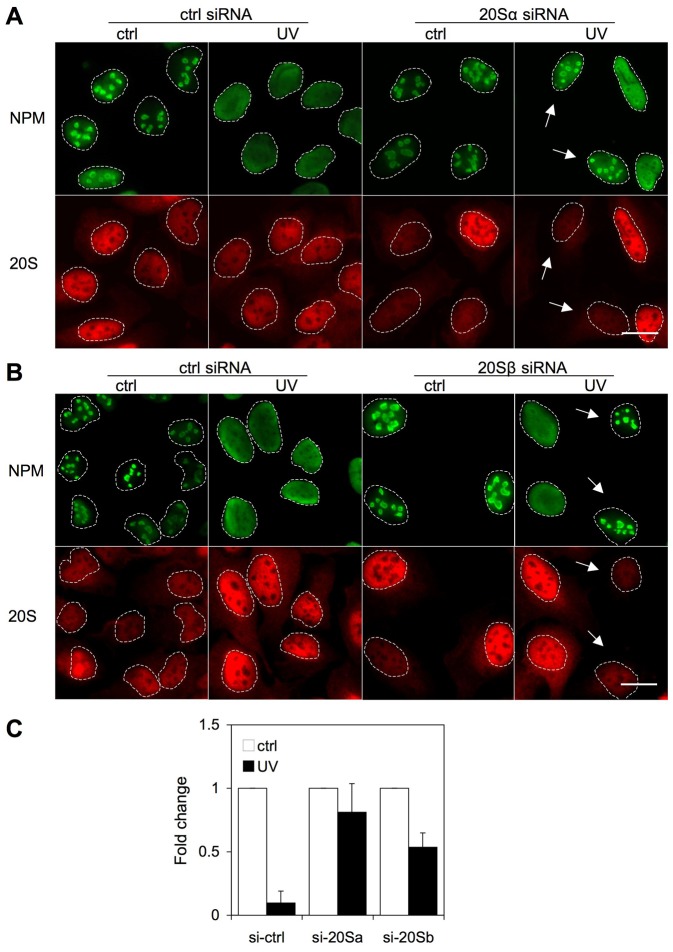
Inhibition of expression of 20S proteasome prevents NPM relocalization after UV radiation. U2OS cells were transfected with specific siRNAs against 20S α (*A*) and 20S β (*B*) subunits and the cells were incubated for 72 hours. The cells were then treated with UV radiation (35 J/m^2^) for 3 hours or left untreated. Cells were fixed and stained for NPM and 20S. *Arrows* indicate 20S silenced cells. *C* Nucleolar areas were quantified from two independent experiments. Scale bars 20 µm.

Finally, despite that there was no apparent indication that UV damage affects NPM proteasomal turnover we proceeded with genetic inhibition of the proteasome, specifically by silencing 20S core subunits responsible for its catalytic activity. We silenced the 20S α and β subunits in U2OS cells using siRNA, and used a random non-targeting siRNA as control. Silencing was confirmed by immunological detection of the 20S subunits (Fig. 7*A and B* and Fig. S7). We treated the cells with UV for 3 hours, fixed and stained the cells for NPM and 20S and quantified NPM nucleolar area. The UV-mediated NPM localization change was clearly inhibited in cells that underwent effective silencing of either 20S α or β subunit (Fig. 7*A, B and C*). This suggests that the proteasome is needed for the observed change in NPM location by UV radiation.

## Discussion

Here we have investigated the regulation of NPM relocation after UV radiation. We found that proteasome inhibition effectively blocks the UV–mediated NPM translocation, but that it was independent of UV damage-activated cellular stress and signaling pathways. In addition to NPM, also other nucleolar GC-proteins were similarly affected and an increase in their nucleoplasmic expression was substantially inhibited by MG132. We found that ubiquitin or ubiquitin recycling were not requisite for these activities, but that the activity of the proteasome was essential for the observed changes in NPM protein localization by UV. However, UV damage did not affect the apparent NPM protein level or half-life, suggesting that NPM by itself is not proteasomally targeted. These findings suggest that the decrease of NPM nucleolar association reflects nucleolar disintegration and nucleoplasmic redistribution of nucleolar proteins and their complexes. In this context, the nucleoplasmic redistribution appears to depend on proteasome-dependent turnover, raising the possibility that NPM is associated with proteins or protein complexes that are subject to proteasome-dependent regulation.

We have shown previously that UV-damage causes widespread dynamic changes in the expression and localization of nucleolar proteins [22]. These changes were documented by quantitative mass spectrometry, cellular imaging and biochemical means, and showed that while a large number of nucleolar proteins were affected by UV, ionizing radiation had a much more limited impact [22]. These findings made us question what underlies the UV-activated drastic changes in nucleolar protein localization. Further, although there are many detailed studies on downstream effects of nucleolar disruption, it is not clear what triggers the localization changes [45]. Since the nucleolus is predominantly formed around active transcription sites [46], disruption of the nucleolus and subsequent protein relocation may represent loss of transcription. However, this view has recently been challenged by demonstration that not all nucleolar proteins are similarly affected, and that even under transcription stress certain proteins accumulate into the nucleolus [22], [28]. Furthermore, UV damage causes a complex activation of cellular signaling networks, including activation of intracellular stress signaling cascades and DNA damage response pathways. Surprisingly, none of the major UV damage-activated pathways, including MEK, JNK and p38 stress signaling routes [19], or DNA damage sensors ATM, ATR and DNA-PK kinase pathways, were prerequisite for the UV-mediated changes in NPM localization. This indicated that the nucleolar response to UV is largely independent of events that relate to the known cellular UV stress responses.

Nucleolar proteins, including NPM are highly mobile [9], [47]. Using photobleaching experiments of UV-treated live cells we show here that the mobility of NPM increases over time, and that NPM is highly diffusible 3 h after UV. These results indicate that analogous to Pol I inhibition, NPM is released from its binding partners like the 60S ribosome following UV damage [37], [48]. In contrast, the mobility of NPM decreases in cells treated with MG132 [25], [27] (Fig. 3). Inhibition of the proteasome function, using specific catalytic inhibitors, effectively led to retention of nucleolar NPM after UV. Although NPM was used as model protein, other GC proteins (NCL, nucleostemin) were similarly affected. The ability of the proteasome inhibitor to inhibit UV-activated localization changes was evident on both endogenous proteins and their fluorescent protein tagged variants. The effect of combination of MG132 with UV treatment on the DFC and FC proteins was more subtle. DFC and FP proteins, represented as UBF and FBL, form nucleolar necklaces and cap structures following transcription inhibition [38] and UV, and were largely unaffected by the combinatory treatment.

A reasonable possibility is that NPM and other GC nucleolar proteins undergo nucleolar translocation due to inhibition of Pol I transcription. From this perspective, it is noteworthy that proteasome inhibition does not affect Pol I transcription, but does inhibit rRNA processing [25], [26]. Here, this was evident by the decrease of the mature 28S RNA transcript following MG132-treatment, while the synthesis of the 47S precursor rRNA was intact. On the other hand, UV damage fully inhibited 47S precursor rRNA transcription. Thus, although the nucleolar expression of NPM, and several other GC proteins was retained following proteasome inhibition, there was no compensatory increase in Pol I transcription, suggesting that the relocation is a cause, rather than effector, of Pol I inhibition.

In addition to its well understood role in protein degradation, ubiquitin contributes to regulation of many cellular processes, like membrane trafficking, protein kinase activation, DNA repair, and chromatin dynamics [49]. Ubiquitin has important roles in DNA damage response and repair, *i.e.* many DNA damage response proteins catalyze ubiquitination or have ubiquitin binding domains [49]. Protein ubiquitination is also involved in UV damage repair [50]. Therefore ubiquitin could contribute to UV–mediated NPM localization changes and its prevention by proteasome inhibition. Further, we have recently shown that proteotoxic stress causes the formation of a protein-RNA aggregate into the nucleolus, and alters nucleolar organization [27]. This aggregate contains nucleoplasmic proteasome target proteins, such as p53 and MDM2, but not nucleolar proteins. Moreover, the formation of the aggregate was alleviated by excess free ubiquitin, suggesting that lack of ubiquitin recycling contributes to the aggregate formation [27]. We therefore manipulated ubiquitin recycling in multiple ways, including increasing the pool of free ubiquitin, overexpressing deubiquitinating enzymes HAUSP and USP36, by inhibiting MDM2, an E3 ligase for p53, and finally by inhibiting the conjugation of ubiquitin by E1 ligase inhibitor. However, none of these affected NPM localization by UV. We conclude that ubiquitin *per se* is unlikely to have a role in UV radiation –mediated NPM translocation. However, we cannot exclude that these effects would be mediated by *e.g.* specific deubiquitinases not tested in our assays, or that an alternative E1, UBA6, could compensate for loss of E1 activity.

Consistent with inhibition of the proteasome catalytic activity by the proteasome inhibitors, we considered that proteasomal degradation is required for NPM relocation by UV. This was despite that we did not observe any change in NPM expression or half-life after UV or after proteasome inhibition, which is unexpected of proteins conventionally considered as proteasomal targets. However, the lack of correlation of protein ubiquitination and increase in protein half-life has been highlighted in a recent large-scale proteomic analysis for ubiquitin-modified proteome [51]. This suggests that ultimately more selective techniques should be in place to assess the potential alterations in protein expression following proteotoxic stress. Notably, most ribosomal proteins have much higher turnover rates in nucleoli as compared to cytoplasm, whereas the turnover of NPM, NCL and GNL3 is invariable [52]. These findings indicate that protein functional associations impact their stability, and that the stabilities may vary greatly in the subcellular compartments. Moreover, ribosomal proteins are highly unstable when Pol I transcription is inhibited by Actinomycin D [53], and following proteotoxic stress, ribosomal proteins accumulate in the nucleoplasm where they are presumed to undergo degradation [54]. These findings suggest that rapid turnover of ribosomal proteins is promoted when Pol I transcription is restricted, like in UV damaged cells. Accordingly, downregulation of proteasomes by specifically silencing the 20S core subunits α and β inhibited the UV–mediated NPM relocation substantiating that the proteasome has an important contribution for the phenotype. Hence, these results suggest the following sequence of events. UV-damage causes repression of Pol I transcription and consequently, nucleoplasmic redistribution of nucleolar proteins or protein complexes. This could affect proteins involved in late ribosome maturation, ribosomal proteins, stress-responsive proteins or RNA-protein complexes that NPM associates with [55]. Loss of functional protein interactions exposes a subset of these proteins to proteasome-dependent degradation whereas other proteins, such as NPM, are retained in the nucleoplasm and display altered mobilities as reflection of changes in their functional associations. This model further suggests that inhibition of the proteasome limits degradation of protein(s) required for stable nucleolar association of NPM.

These findings provide an intriguing insight for the relevance of the proteasome activity in nucleolar protein fates and localization following nucleolar stress. They substantiate the significance of the proteasome in quality control of nucleolar proteins, rRNA and the ribosomes and the tight coupling of Pol I transcription and proteasome function. In future it will be pertinent to resolve how the ubiquitin-proteasome function is involved in Pol I transcription, rRNA processing and ribosome assembly and how it is affected in cellular stress.

## Materials and Methods

### Plasmids

NPM-ECGFP fusion protein was generated as described [22]. USP36-FLAG was obtained from Origene. HA-Ub-wt/pcDNA3 was a kind gift from Dr I. Dikic (Goethe University, Frankfurt, Germany [56]), and pCIneo-HAUSP-Flag (USP7) vector was kindly provided by Dr B. Vogelstein (Johns Hopkins University, Baltimore, MD, USA [57]).

### Cell Culture, Chemicals, Treatments and Transfections

WS1 human skin fibroblasts (CRL-1502, ATCC) were maintained in DMEM supplemented with 10% FCS, non-essential amino acids and penicillin-streptomycin. U2OS human osteosarcoma cells (HTB-96, ATCC) were maintained in DMEM supplemented with 15% FCS. A375 human melanoma cells (CRL-1619, ATCC) and HeLa cervical adenocarcinoma cells (CCL-2, ATCC) were maintained in DMEM supplemented with 10% FCS. Stable U2OS cell lines (NPM-ECGFP and USP36-FLAG) were generated by transfecting the constructs by lipofection (Lipofectamine, Invitrogen), selection in the presence of G418, and isolation of single cell colonies. Stable clones were maintained in the presence of G418. All cells were maintained at +37°C in a humidified atmosphere containing 5% CO_2_. Chemicals used were U0126, SB203580, wortmannin, KU55933 and lactacystin (Calbiochem), SP600125 (A. G. Scientific), NU7441 (Santa Cruz), MG132 (Enzo/Biomol), UBE-41 (Biogenova) and Nutlin-3 (Alexis Biochemicals). All other cell culture reagents were obtained from Gibco-BRL and Sigma. Cells were treated with UVC using 254 nm UVC light bulbs (Stratalinker).

### Fluorescent recovery after photobleaching

U2OS cells plated on Lab-Tek chambers (Nalge Nunc International) were transfected with NPM-ECGFP by lipofection (Lipofectamine, Invitrogen) or U2OS cells stably expressing NPM-ECGFP were used [22]. The following day the growth medium was replaced with DMEM without phenol red (Gibco-BRL). The cells were maintained at +37°C using a heating stage or an incubator during the imaging. Photobleaching and imaging was performed using either Zeiss LSM510 META confocal microscope equipped with 458 nm Argon laser at 85% output (7.3 A) and Plan-Neofluar 40×/1.3NA Oil objective with 100% laser power during the bleaching and at 2% during the imaging (Fig. 1 and Fig. S1), or Zeiss LSM510 DUO equipped with 488 nm Argon laser at 50% output (6.1 A) and Plan-Apochromat 40×/1.3NA Oil objective with 100% laser power during the bleaching and at 1% during the imaging (Fig. 3). ROI (region of interest) was determined as single nucleolus, which was bleached after 3 pre-scans with 30 iterations. 97 or 297 post-bleach images were captured for Figures 1 and 3, respectively. Total intensity of the nucleus and background ROIs were recorded simultaneously. Fluorescent intensities were measured by LSM 510 Physiology Software. Raw data was exported into Microsoft Office Excel software to perform image analysis calculations according to [58]. Background fluorescent values were subtracted from each image, the values were corrected to compensate the decrease in the total intensity caused by scanning, and the results were normalized. Mobile fractions were calculated as described in [58]:
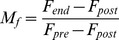



where F_end_ is the fluorescent intensity after reaching the plateau (average of the last 20 scans), F_post_ is the fluorescent intensity immediately after the bleach, and F_pre_ before the bleach (average of 3 pre-scans). Eight to nine cells were analyzed from at least two independent experiments (Fig. 1) or five to eight cells from three to four independent experiments (Fig. 3). Recovery half times were calculated according to [58]:
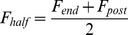


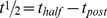



### Immunofluorescence and Image Analysis

Cells were fixed with 3.5% paraformaldehyde followed by permeabilization with 0.5% NP-40. The following primary antibodies were used: mouse anti-NPM (Zymed/Invitrogen), rabbit anti-nucleostemin (GNL3, H-270, Santa Cruz), mouse anti-NCL (Abcam), rabbit anti-UBF (H-300, Santa Cruz), rabbit anti-FBL (Abcam), rabbit anti-HA (Y-11, Santa Cruz), rabbit anti-FLAG (Sigma), rabbit anti-proteasome 20S core subunits (PW8155, Enzo/Biomol) and rabbit anti-p53 (7F5, Cell Signaling Technologies). Antibodies were detected with secondary antibodies conjugated to Alexa 488 or 594 (Molecular Probes) and nuclei were counterstained with either Hoechst 33258 or 33342. The fluorochromes were visualized with Zeiss Axioplan 2 Imaging MOT (Jena, Germany) epifluorescence microscope equipped with 20×/0.5NA Plan-Neofluar objective and Chroma 31000v2, Chroma 41001, and Chroma 41004 filters. Images were captured with Zeiss AxioCam HRm 14-bit grayscale CCD camera and AxioVision program version 4.6 and 4.7. Confocal imaging was performed with Zeiss LSM510 META (Jena, Germany) microscope equipped with 63/1.25 NA Plan-Neofluar objective, and diode and HeNe lasers.

Images were quantified by Fiji/ImageJ-software. For quantification of NPM signal intensity, cells were co-stained for NPM and UBF. Nucleolar area was determined by UBF staining (UBF mask) because UBF and NPM mask areas showed good overlap (87%) indicating that UBF, which is retained in the nucleolar area even after UV radiation, could be used as a surrogate marker for the nucleolus. NPM mean intensity was then calculated from the UBF mask area. Nucleoplasmic NPM intensity was determined by first defining the nuclear area according to Hoechst staining (DNA mask), followed by subtracting the UBF mask area. The remnant was designated as nucleoplasmic area. NPM mean intensity in the nucleoplasmic area was then determined, and NPM nucleolar/nucleoplasmic ratios were calculated. NPM intensity ratios were calculated from three independent experiments, each of which contained two fields per treatment (N ≥ 140 cells). Image analysis in Fig. 6 and 7 was conducted using FrIDA designed for the analysis of RGB color image datasets [59]. Hue saturation and brightness range were defined for transfected and non-transfected cells and normalized to DNA. An average of 200 non-transfected cells and 20 transfected cells was quantified from three-five fields for each experiment.

*P*-values were calculated by Student's two-tailed T test, *P<0.05; **P<0.01; ***P<0.001.

### Western Blotting

To obtain cellular lysates, cells were scraped, solubilized in RIPA buffer (50 mM Tris–HCl (pH 7.5), 150 mM NaCl, 1% NP-40, 0.1% sodium dodecyl sulfate, 1% sodium deoxycholate) supplemented with Complete Protease Inhibitor Tablets (Roche) and sonicated in ice-cold water bath for 15 min or briefly by peak sonicator followed by centrifugation at 13,000 rpm for 15 min. Protein concentration was determined using Bio-Rad Bradford protein assay (Bio-Rad, Hercules, CA). Equal amounts of protein were loaded into 9% SDS-PAGE and transferred into nitrocellulose membrane (Trans-Blot, Transfer Medium, Bio-Rad). Immunoblotting was carried out using mouse anti-NPM (Zymed/Invitrogen), rabbit anti-GLN3 (Santa Cruz), mouse anti-NCL (Abcam), mouse anti-UBF (F-9, Santa Cruz), rabbit anti-FBL (Abcam), mouse anti-α-tubulin (clone B-5-1-2, Sigma), DO-1 (p53) and rabbit anti-proteasome 20S core subunits (PW8155, Enzo/Biomol) antibodies followed by incubation with secondary antibodies conjugated either directly to horseradish peroxidase or via biotin-streptavidin, after which the signals were detected using enhanced chemiluminescence (ECL, Amersham Life Sciences).

### Ethynyl Uridine –labeling

Cells were labeled with 1 mM ethynyl uridine (EU, Invitrogen). Cells were fixed and EU signal was detected using Click-iT RNA Alexa Fluor® 488 Imaging Kit (Invitrogen) according to manufacturer's protocol. To quantify incorporation of EU, nuclei were first identified by Hoechst staining and the EU mean intensity values were collected from the nuclear areas from two independent experiments. N = 50–70 cells were analyzed in each experiment. *P*-values were calculated using Student's two-tailed T test.

### Metabolic Labeling

^3^H-labeled uridine (Perkin Elmer, final concentration 2–4 µCi/mL) was incubated with the cells for the last 1–2 hours. RNA was extracted by NucleoSpin RNA II kit (Macherey-Nagel) and RNA concentrations were measured with NanoDrop. Equal amounts of RNA was separated on 1% formaldehyde-agarose gel and transferred onto Hybond-N+ −filter (Amersham). The filter was cross-linked and sprayed with EN^3^HANCE (Perkin Elmer). Autoradiographs were developed 2 to 7 days later.

### RNAi

U2OS cells were plated on coverslips and transfected with specific siRNAs either at the time of plating or the following day. The following siRNAs were used: Hs_PSMA3_5 FlexiTube siRNA (SI00301434, Qiagen) for 20Sα and Hs_PSMB1_2 FlexiTube siRNA (SI00301455, Qiagen) for 20Sβ.

## Supporting Information

Figure S1 **NPM nucleoplasmic mobility is high following UV radiation.** *A* U2OS cells were transiently transfected with NPM-ECGFP and were treated with UVC (35 J/m^2^) for 6 hours. FRAP analysis was performed on nucleoplasm as indicated by ROI (*red* circle). Following photobleaching images were captured every 1 s for 100 s. Representative images are shown. Scale bar 10 µm. *B* Averages of normalized intensities and the mobile fraction from at least two independent experiments is shown. Error bars, SD. *N* = 10 cells.
(TIF)

Figure S2 **Inhibition of DNA damage or UV-activated cell stress signaling pathways do not affect UV-mediated NPM relocalization.** U2OS cells were treated with inhibitors targeting UV-activated cellular signaling (U0126 10 µM for MEK, SB203580 20 µM for p38 and SP600125 100 µM for JNK), DNA damage signaling (KU55933 10 µM for ATM, wortmannin 100 µM for ATM/ATR and NU7441 10 µM for DNA-PK) and proteasome (MG132 10 µM) or left untreated. One hour later the cells were exposed to UV radiation (35 J/m^2^) or left untreated. Cells were fixed after 3 hours and stained for NPM. Scale bar, 50 µm.
(TIF)

Figure S3 **NPM relocalization is not antibody-specific and NPM protein levels remain constant in different cell lines.** *A* U2OS cells stably expressing NPM-ECGFP were treated with MG132 or left untreated. After 2 hours the cells were treated with UV (35 J/m^2^) and incubated for 6 hours. Scale bar 20 µm. *B* HeLa and U2OS cells were pretreated with MG132 and UV (35 J/m^2^) as shown. After 3 hours cells were lysed with RIPA buffer. Equal amounts of total protein were separated by SDS-PAGE and immunoblotted for NPM. Tubulin was used as a loading control.
(TIF)

Figure S4 **NPM half-life is unaltered following UV damage.** *A* and *B*, U2OS cells were treated with UV (35 J/m^2^) and incubated in the presence or absence of cycloheximide (CHX, 50 µg/ml) for the indicated times. Cell lysates were prepared and analyzed by immunoblotting for NPM and GAPDH as control. *C*, U2OS cells were treated with UV (35 J/m^2^) in the presence or absence of cycloheximide (50 µg/ml) and incubated for 3 h. Fixed cells were stained for NPM (*red*) and DNA (*blue*). *D*, U2OS cells were treated with UV (35 J/m^2^) in the presence or absence of α-amanitin (25 µg/ml) and incubated for 3 h. Fixed cells were stained for NPM (*red*) and DNA (*blue*).
(TIF)

Figure S5 **Nutlin-3 does not affect NPM redistribution after UV.** U2OS cells were treated with either Nutlin-3 (10 µM) or UV (35 J/m^2^), or pretreated with Nutlin-3 for 1 hour followed by UV treatment and incubated for 3 hours, or left untreated (control). The cells were fixed and stained for NPM and p53. Scale bar, 20 µm.
(TIF)

Figure S6 **UbE1 inhibitor induces p53 response.** WS1 cells were treated with UV (35 J/m^2^) or UbE1 inhibitor (10 µM) and incubated for 19 hours or left untreated. The cells were fixed and stained for p53.
(TIF)

Figure S7 **Silencing of 20S proteasome.** HeLa cells were transfected with specific siRNAs against 20S α proteasome and the cells were incubated for 72 hours. Equal amounts of total protein were separated by SDS-PAGE and immunoblotted for p53 and 20S. Tubulin was used as a loading control.
(TIF)

## References

[1] Grummt I (2003) Life on a planet of its own: Regulation of RNA polymerase I transcription in the nucleolus. Genes Dev 17 1691–1702: 10.1101/gad.1098503R. 10.1101/gad.1098503R 12865296

[2] Russell J, Zomerdijk JC (2006) The RNA polymerase I transcription machinery. Biochem Soc Symp (73): 203–216. 10.1042/bss0730203 PMC3858827 16626300

[3] Fatica A, Tollervey D (2002) Making ribosomes. Curr Opin Cell Biol 14: 313–318. 12067653 10.1016/s0955-0674(02)00336-8

[4] Moss T, Langlois F, Gagnon-Kugler T, Stefanovsky V (2007) A housekeeper with power of attorney: The rRNA genes in ribosome biogenesis. Cell Mol Life Sci 64: 29–49 10.1007/s00018-006-6278-1. 17171232 10.1007/s00018-006-6278-1 PMC11136161

[5] Moss T (2004) At the crossroads of growth control; making ribosomal RNA. Curr Opin Genet Dev 14: 210–217 10.1016/j.gde.2004.02.005. 15196469 10.1016/j.gde.2004.02.005

[6] Leary DJ, Huang S (2001) Regulation of ribosome biogenesis within the nucleolus. FEBS Lett 509: 145–150. 11741579 10.1016/s0014-5793(01)03143-x

[7] Ahmad Y, Boisvert FM, Gregor P, Cobley A, Lamond AI (2009) NOPdb: Nucleolar proteome database--2008 update. Nucleic Acids Res 37: D181–4 10.1093/nar/gkn804. 18984612 10.1093/nar/gkn804 PMC2686505

[8] Olson MO, Dundr M (2005) The moving parts of the nucleolus. Histochem Cell Biol 123 203–216: 10.1007/s00418–005-0754-9. 10.1007/s00418-005-0754-9 15742198

[9] Dundr M, Hoffmann-Rohrer U, Hu Q, Grummt I, Rothblum LI, et al (2002) A kinetic framework for a mammalian RNA polymerase in vivo. Science 298: 1623–1626 10.1126/science.1076164. 12446911 10.1126/science.1076164

[10] Rubbi CP, Milner J (2003) Disruption of the nucleolus mediates stabilization of p53 in response to DNA damage and other stresses. EMBO J 22: 6068–6077 10.1093/emboj/cdg579. 14609953 10.1093/emboj/cdg579 PMC275437

[11] Olson MO (2004) Sensing cellular stress: Another new function for the nucleolus? Sci STKE 224: pe10 10.1126/stke.2242004pe10. 10.1126/stke.2242004pe10 15026578

[12] Mayer C, Grummt I (2005) Cellular stress and nucleolar function. Cell Cycle 4: 1036–1038. 16205120 10.4161/cc.4.8.1925

[13] Stark LA, Taliansky M (2009) Old and new faces of the nucleolus. workshop on the nucleolus and disease. EMBO Rep 10: 35–40 10.1038/embor.2008.230. 19079131 10.1038/embor.2008.230 PMC2613212

[14] Pederson T (2011) The nucleolus. Cold Spring Harb Perspect Biol 3 10.1101/cshperspect.a000638. 10.1101/cshperspect.a000638 PMC3039934 21106648

[15] Pederson T, Tsai RY (2009) In search of nonribosomal nucleolar protein function and regulation. J Cell Biol 184: 771–776 10.1083/jcb.200812014. 19289796 10.1083/jcb.200812014 PMC2699146

[16] Lindstrom MS (2009) Emerging functions of ribosomal proteins in gene-specific transcription and translation. Biochem Biophys Res Commun 379: 167–170 10.1016/j.bbrc.2008.12.083. 19114035 10.1016/j.bbrc.2008.12.083

[17] Kurki S, Peltonen K, Latonen L, Kiviharju TM, Ojala PM, et al (2004) Nucleolar protein NPM interacts with HDM2 and protects tumor suppressor protein p53 from HDM2-mediated degradation. Cancer Cell 5: 465–475. 15144954 10.1016/s1535-6108(04)00110-2

[18] Ravanat JL, Douki T, Cadet J (2001) Direct and indirect effects of UV radiation on DNA and its components. J Photochem Photobiol B 63: 88–102. 11684456 10.1016/s1011-1344(01)00206-8

[19] Herrlich P, Karin M, Weiss C (2008) Supreme EnLIGHTenment: Damage recognition and signaling in the mammalian UV response. Mol Cell 29: 279–290 10.1016/j.molcel.2008.01.001. 18280234 10.1016/j.molcel.2008.01.001 PMC2714880

[20] Zhang Y, Lu H (2009) Signaling to p53: Ribosomal proteins find their way. Cancer Cell 16: 369–377 10.1016/j.ccr.2009.09.024. 19878869 10.1016/j.ccr.2009.09.024 PMC4369769

[21] Shcherbik N, Pestov DG (2010) Ubiquitin and ubiquitin-like proteins in the nucleolus: Multitasking tools for a ribosome factory. Genes Cancer 1: 681–689 10.1177/1947601910381382. 21113400 10.1177/1947601910381382 PMC2991155

[22] Moore HM, Bai B, Boisvert FM, Latonen L, Rantanen V, et al (2011) Quantitative proteomics and dynamic imaging of the nucleolus reveal distinct responses to UV and ionizing radiation. Mol Cell Proteomics 10 M111.009241. 10.1074/mcp.M111.009241. 10.1074/mcp.M111.009241 PMC3205868 21778410

[23] Ye Y, Rape M (2009) Building ubiquitin chains: E2 enzymes at work. Nat Rev Mol Cell Biol 10: 755–764 10.1038/nrm2780. 19851334 10.1038/nrm2780 PMC3107738

[24] Finley D (2009) Recognition and processing of ubiquitin-protein conjugates by the proteasome. Annu Rev Biochem 78: 477–513 10.1146/annurev.biochem.78.081507.101607. 19489727 10.1146/annurev.biochem.78.081507.101607 PMC3431160

[25] Stavreva DA, Kawasaki M, Dundr M, Koberna K, Muller WG, et al (2006) Potential roles for ubiquitin and the proteasome during ribosome biogenesis. Mol Cell Biol 26: 5131–5145 10.1128/MCB.02227-05. 16782897 10.1128/MCB.02227-05 PMC1489179

[26] Burger K, Muhl B, Harasim T, Rohrmoser M, Malamoussi A, et al (2010) Chemotherapeutic drugs inhibit ribosome biogenesis at various levels. J Biol Chem 285( 12416–12425 10.1074/jbc.M109.074211. 10.1074/jbc.M109.074211 PMC2852979 20159984

[27] Latonen L, Moore HM, Bai B, Jaamaa S, Laiho M (2011) Proteasome inhibitors induce nucleolar aggregation of proteasome target proteins and polyadenylated RNA by altering ubiquitin availability. Oncogene 30: 790–805 10.1038/onc.2010.469. 20956947 10.1038/onc.2010.469

[28] Andersen JS, Lam YW, Leung AK, Ong SE, Lyon CE, et al (2005) Nucleolar proteome dynamics. Nature 433: 77–83 10.1038/nature03207. 15635413 10.1038/nature03207

[29] Fujii K, Kitabatake M, Sakata T, Miyata A, Ohno M (2009) A role for ubiquitin in the clearance of nonfunctional rRNAs. Genes Dev 23: 963–974 10.1101/gad.1775609. 19390089 10.1101/gad.1775609 PMC2675866

[30] Finley D, Bartel B, Varshavsky A (1989) The tails of ubiquitin precursors are ribosomal proteins whose fusion to ubiquitin facilitates ribosome biogenesis. Nature 338: 394–401 10.1038/338394a0. 2538753 10.1038/338394a0

[31] Redman KL, Rechsteiner M (1989) Identification of the long ubiquitin extension as ribosomal protein S27a. Nature 338: 438–440 10.1038/338438a0. 2538756 10.1038/338438a0

[32] Mattsson K, Pokrovskaja K, Kiss C, Klein G, Szekely L (2001) Proteins associated with the promyelocytic leukemia gene product (PML)-containing nuclear body move to the nucleolus upon inhibition of proteasome-dependent protein degradation. Proc Natl Acad Sci U S A 98: 1012–1017 10.1073/pnas.031566998. 11158586 10.1073/pnas.031566998 PMC14700

[33] Arabi A, Rustum C, Hallberg E, Wright AP (2003) Accumulation of c-myc and proteasomes at the nucleoli of cells containing elevated c-myc protein levels. J Cell Sci 116: 1707–1717. 12665552 10.1242/jcs.00370

[34] Scharf A, Rockel TD, von Mikecz A (2007) Localization of proteasomes and proteasomal proteolysis in the mammalian interphase cell nucleus by systematic application of immunocytochemistry. Histochem Cell Biol 127: 591–601 10.1007/s00418-006-0266-2. 17205305 10.1007/s00418-006-0266-2

[35] Boyd MT, Vlatkovic N, Rubbi CP (2011) The nucleolus directly regulates p53 export and degradation. J Cell Biol 194: 689–703 10.1083/jcb.201105143. 21893597 10.1083/jcb.201105143 PMC3171122

[36] Endo A, Matsumoto M, Inada T, Yamamoto A, Nakayama KI, et al (2009) Nucleolar structure and function are regulated by the deubiquitylating enzyme USP36. J Cell Sci 122: 678–686 10.1242/jcs.044461. 19208757 10.1242/jcs.044461

[37] Chen D, Huang S (2001) Nucleolar components involved in ribosome biogenesis cycle between the nucleolus and nucleoplasm in interphase cells. J Cell Biol 153: 169–176. 11285283 10.1083/jcb.153.1.169 PMC2185520

[38] Shav-Tal Y, Blechman J, Darzacq X, Montagna C, Dye BT, et al (2005) Dynamic sorting of nuclear components into distinct nucleolar caps during transcriptional inhibition. Mol Biol Cell 16: 2395–2413 10.1091/mbc.E04-11-0992. 15758027 10.1091/mbc.E04-11-0992 PMC1087244

[39] Latonen L, Laiho M (2005) Cellular UV damage responses--functions of tumor suppressor p53. Biochim Biophys Acta 1755: 71–89. 15921859 10.1016/j.bbcan.2005.04.003

[40] Cioce M, Boulon S, Matera AG, Lamond AI (2006) UV-induced fragmentation of cajal bodies. J Cell Biol 175: 401–413 10.1083/jcb.200604099. 17088425 10.1083/jcb.200604099 PMC2064518

[41] Endo A, Kitamura N, Komada M (2009) Nucleophosmin/B23 regulates ubiquitin dynamics in nucleoli by recruiting deubiquitylating enzyme USP36. J Biol Chem 284: 27918–27923 10.1074/jbc.M109.037218. 19679658 10.1074/jbc.M109.037218 PMC2788843

[42] Huang M, Itahana K, Zhang Y, Mitchell BS (2009) Depletion of guanine nucleotides leads to the Mdm2-dependent proteasomal degradation of nucleostemin. Cancer Res 69: 3004–3012 10.1158/0008-5472.CAN-08-3413. 19318567 10.1158/0008-5472.CAN-08-3413 PMC4568828

[43] Lo D, Dai MS, Sun XX, Zeng SX, Lu H (2012) Ubiquitin- and MDM2 E3 ligase-independent proteasomal turnover of nucleostemin in response to GTP depletion. J Biol Chem 287: 10013–10020 10.1074/jbc.M111.335141. 22318725 10.1074/jbc.M111.335141 PMC3323031

[44] Yang Y, Kitagaki J, Dai RM, Tsai YC, Lorick KL, et al (2007) Inhibitors of ubiquitin-activating enzyme (E1), a new class of potential cancer therapeutics. Cancer Res 67: 9472–9481 10.1158/0008-5472.CAN-07-0568. 17909057 10.1158/0008-5472.CAN-07-0568

[45] Boulon S, Westman BJ, Hutten S, Boisvert FM, Lamond AI (2010) The nucleolus under stress. Mol Cell 40: 216–227 10.1016/j.molcel.2010.09.024. 20965417 10.1016/j.molcel.2010.09.024 PMC2987465

[46] Melese T, Xue Z (1995) The nucleolus: An organelle formed by the act of building a ribosome. Curr Opin Cell Biol 7: 319–324. 7662360 10.1016/0955-0674(95)80085-9

[47] Borer RA, Lehner CF, Eppenberger HM, Nigg EA (1989) Major nucleolar proteins shuttle between nucleus and cytoplasm. Cell 56: 379–390. 2914325 10.1016/0092-8674(89)90241-9

[48] Louvet E, Junera HR, Le Panse S, Hernandez-Verdun D (2005) Dynamics and compartmentation of the nucleolar processing machinery. Exp Cell Res 304: 457–470 10.1016/j.yexcr.2004.11.018. 15748891 10.1016/j.yexcr.2004.11.018

[49] Chen ZJ, Sun LJ (2009) Nonproteolytic functions of ubiquitin in cell signaling. Mol Cell 33: 275–286 10.1016/j.molcel.2009.01.014. 19217402 10.1016/j.molcel.2009.01.014

[50] Bergink S, Jaspers NG, Vermeulen W (2007) Regulation of UV-induced DNA damage response by ubiquitylation. DNA Repair (Amst) 6: 1231–1242 10.1016/j.dnarep.2007.01.012. 17363340 10.1016/j.dnarep.2007.01.012

[51] Kim W, Bennett EJ, Huttlin EL, Guo A, Li J, et al (2011) Systematic and quantitative assessment of the ubiquitin-modified proteome. Mol Cell 44: 325–340 10.1016/j.molcel.2011.08.025. 21906983 10.1016/j.molcel.2011.08.025 PMC3200427

[52] Boisvert FM, Ahmad Y, Gierlinski M, Charriere F, Lamont D, et al (2012) A quantitative spatial proteomics analysis of proteome turnover in human cells. Mol Cell Proteomics 11 M111.011429. 10.1074/mcp.M111.011429. 10.1074/mcp.M111.011429 PMC3316722 21937730

[53] Warner JR (1977) In the absence of ribosomal RNA synthesis, the ribosomal proteins of HeLa cells are synthesized normally and degraded rapidly. J Mol Biol 115: 315–333. 592369 10.1016/0022-2836(77)90157-7

[54] Lam YW, Lamond AI, Mann M, Andersen JS (2007) Analysis of nucleolar protein dynamics reveals the nuclear degradation of ribosomal proteins. Curr Biol 17: 749–760 10.1016/j.cub.2007.03.064. 17446074 10.1016/j.cub.2007.03.064 PMC1885954

[55] Colombo E, Alcalay M, Pelicci PG (2011) Nucleophosmin and its complex network: A possible therapeutic target in hematological diseases. Oncogene 30: 2595–2609 10.1038/onc.2010.646; 10.1038/onc.2010.646. 21278791 10.1038/onc.2010.646

[56] Haglund K, Sigismund S, Polo S, Szymkiewicz I, Di Fiore PP, et al (2003) Multiple monoubiquitination of RTKs is sufficient for their endocytosis and degradation. Nat Cell Biol 5: 461–466 10.1038/ncb983. 12717448 10.1038/ncb983

[57] Cummins JM, Rago C, Kohli M, Kinzler KW, Lengauer C, et al (2004) Tumour suppression: Disruption of HAUSP gene stabilizes p53. Nature 428: 1 p following 486 10.1038/nature02501. 10.1038/nature02501 15058298

[58] Bancaud A, Huet S, Rabut G, Ellenberg J (2010) Fluorescence perturbation techniques to study mobility and molecular dynamics of proteins in live cells: FRAP, photoactivation, photoconversion, and FLIP. Cold Spring Harb Protoc 2010 pdb.top90. 10.1101/pdb.top90. 10.1101/pdb.top90 21123431

[59] Cornish T, Morgan J, Gurel B, De Marzo AM (2008) FrIDA: An open source framework for image dataset analysis. Arch Pathol Lab Med 132: 856.

