# Supplementary figures and images for: Proteasome Activity Influences UV-Mediated Subnuclear Localization Changes of NPM
Source: PLoS One. 2013 Mar 12;8(3):e59096. doi: 10.1371/journal.pone.0059096 (PMC3595268; doi:10.1371/journal.pone.0059096)

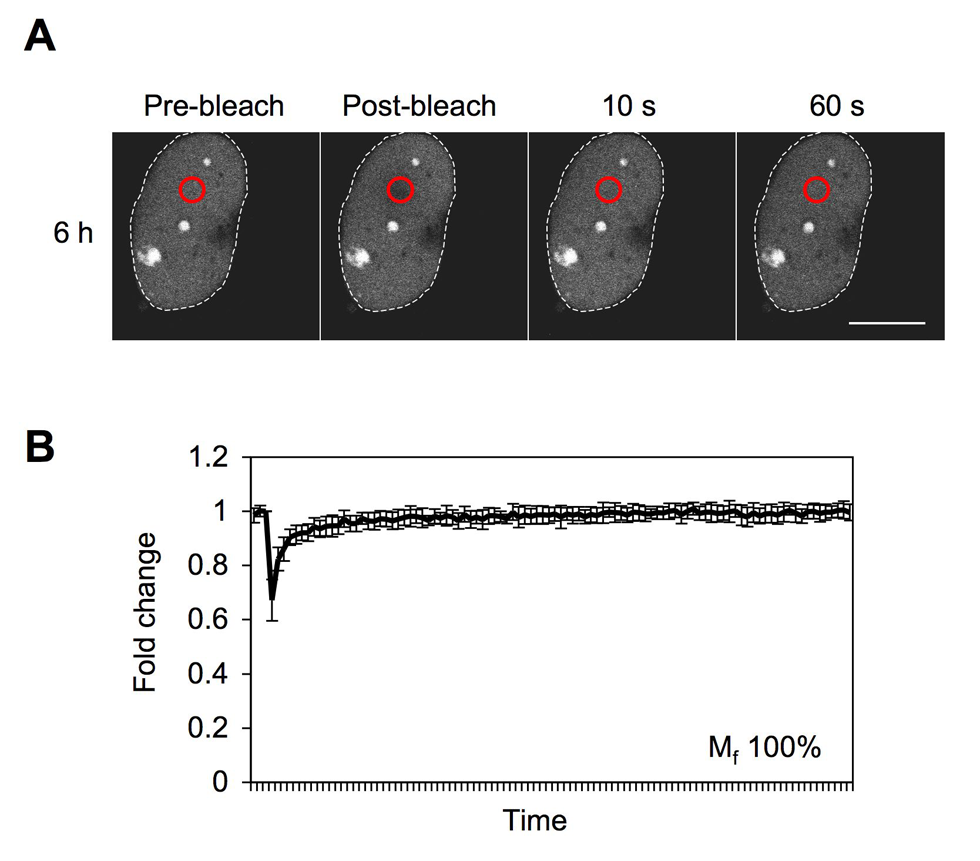

Supplement: Figure S1 — NPM nucleoplasmic mobility is high following UV radiation. A U2OS cells were transiently transfected with NPM-ECGFP and were treated with UVC (35 J/m2) for 6 hours. FRAP analysis was performed on nucleoplasm as indicated by ROI (red circle). Following photobleaching images were captured every 1 s for 100 s. Representative images are shown. Scale bar 10 µm. B Averages of normalized intensities and the mobile fraction from at least two independent experiments is shown. Error bars, SD. N = 10 cells. (TIF) [file pone.0059096.s001.tif]

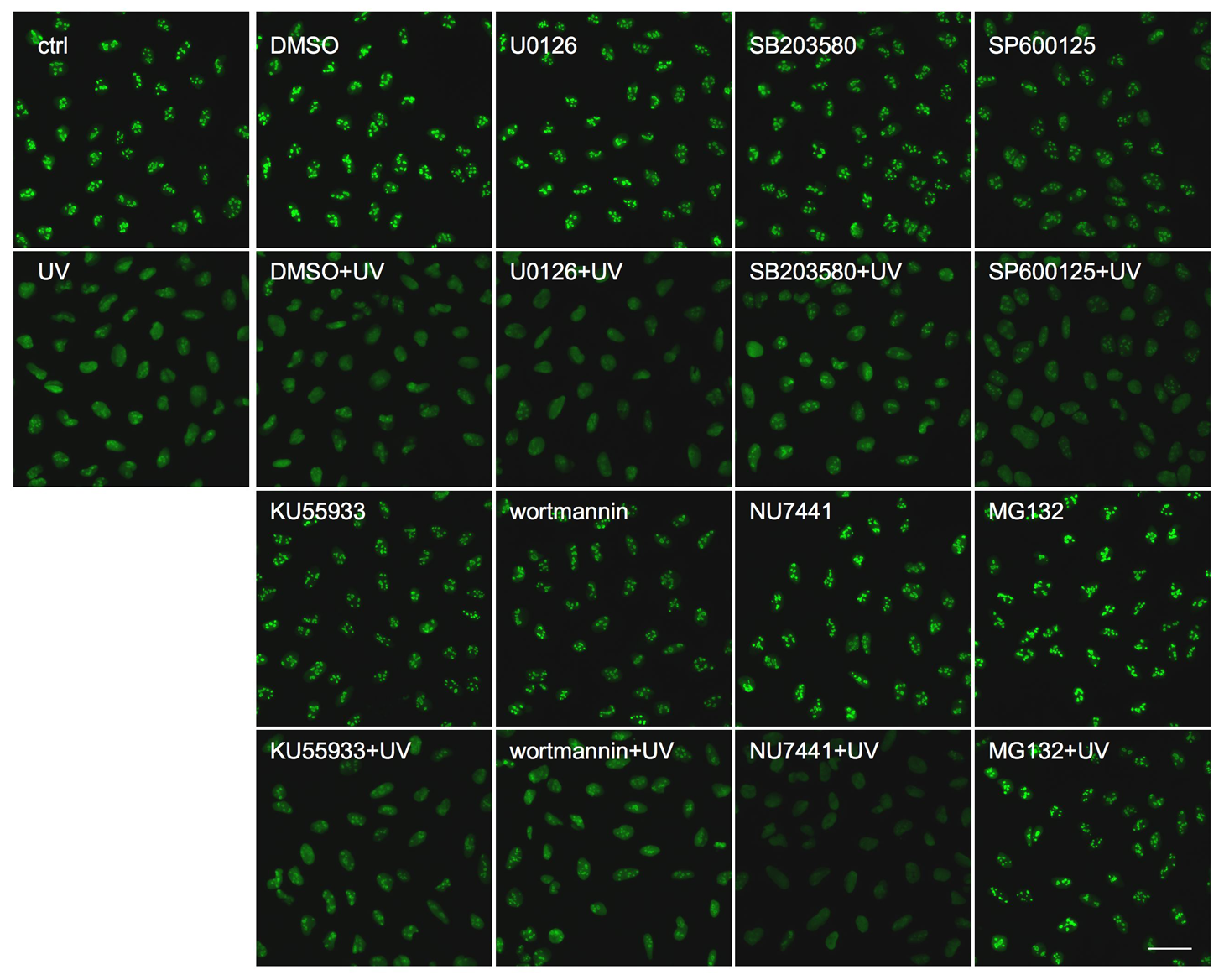

Supplement: Figure S2 — Inhibition of DNA damage or UV-activated cell stress signaling pathways do not affect UV-mediated NPM relocalization. U2OS cells were treated with inhibitors targeting UV-activated cellular signaling (U0126 10 µM for MEK, SB203580 20 µM for p38 and SP600125 100 µM for JNK), DNA damage signaling (KU55933 10 µM for ATM, wortmannin 100 µM for ATM/ATR and NU7441 10 µM for DNA-PK) and proteasome (MG132 10 µM) or left untreated. One hour later the cells were exposed to UV radiation (35 J/m2) or left untreated. Cells were fixed after 3 hours and stained for NPM. Scale bar, 50 µm. (TIF) [file pone.0059096.s002.tif]

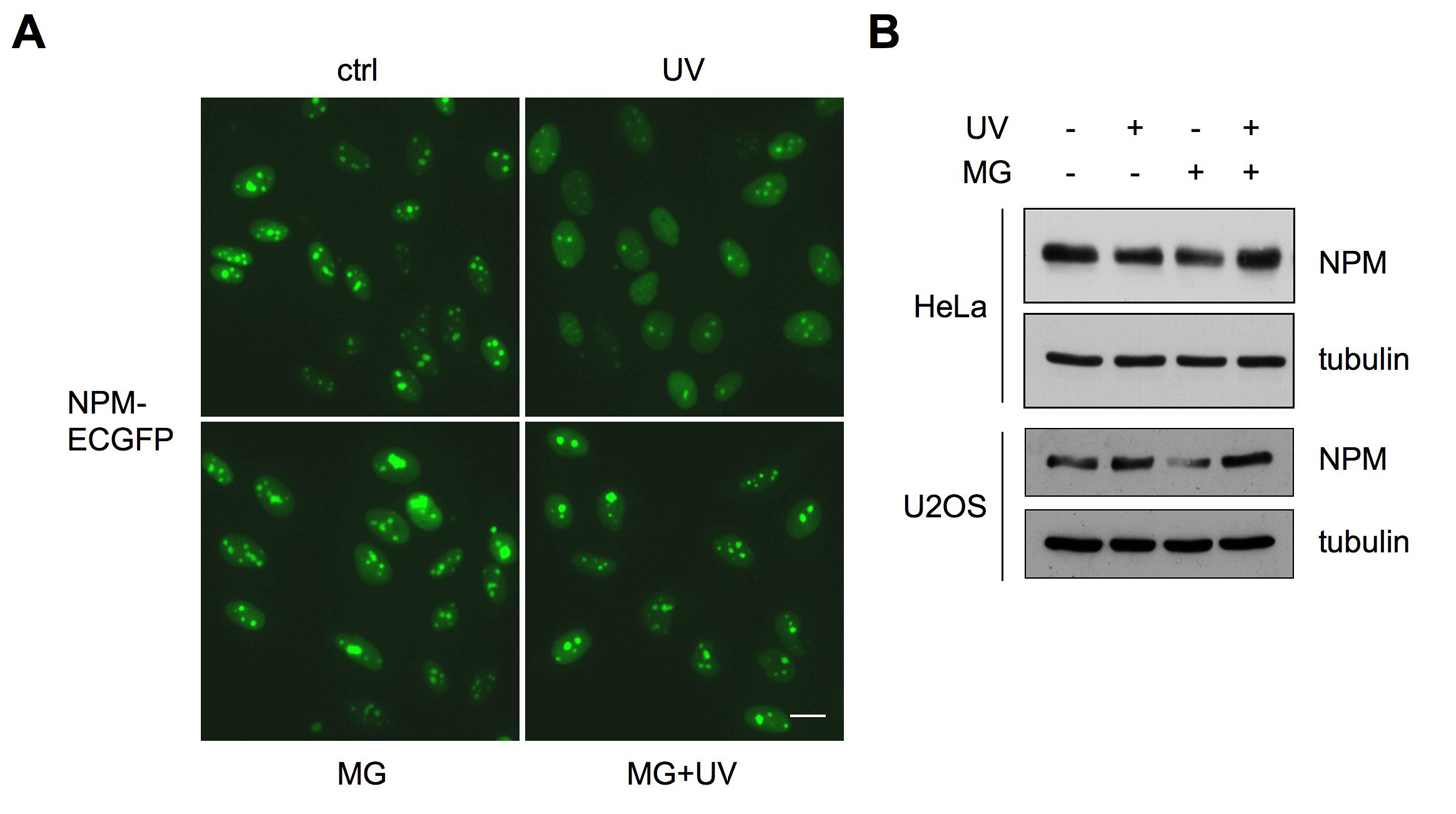

Supplement: Figure S3 — NPM relocalization is not antibody-specific and NPM protein levels remain constant in different cell lines. A U2OS cells stably expressing NPM-ECGFP were treated with MG132 or left untreated. After 2 hours the cells were treated with UV (35 J/m2) and incubated for 6 hours. Scale bar 20 µm. B HeLa and U2OS cells were pretreated with MG132 and UV (35 J/m2) as shown. After 3 hours cells were lysed with RIPA buffer. Equal amounts of total protein were separated by SDS-PAGE and immunoblotted for NPM. Tubulin was used as a loading control. (TIF) [file pone.0059096.s003.tif]

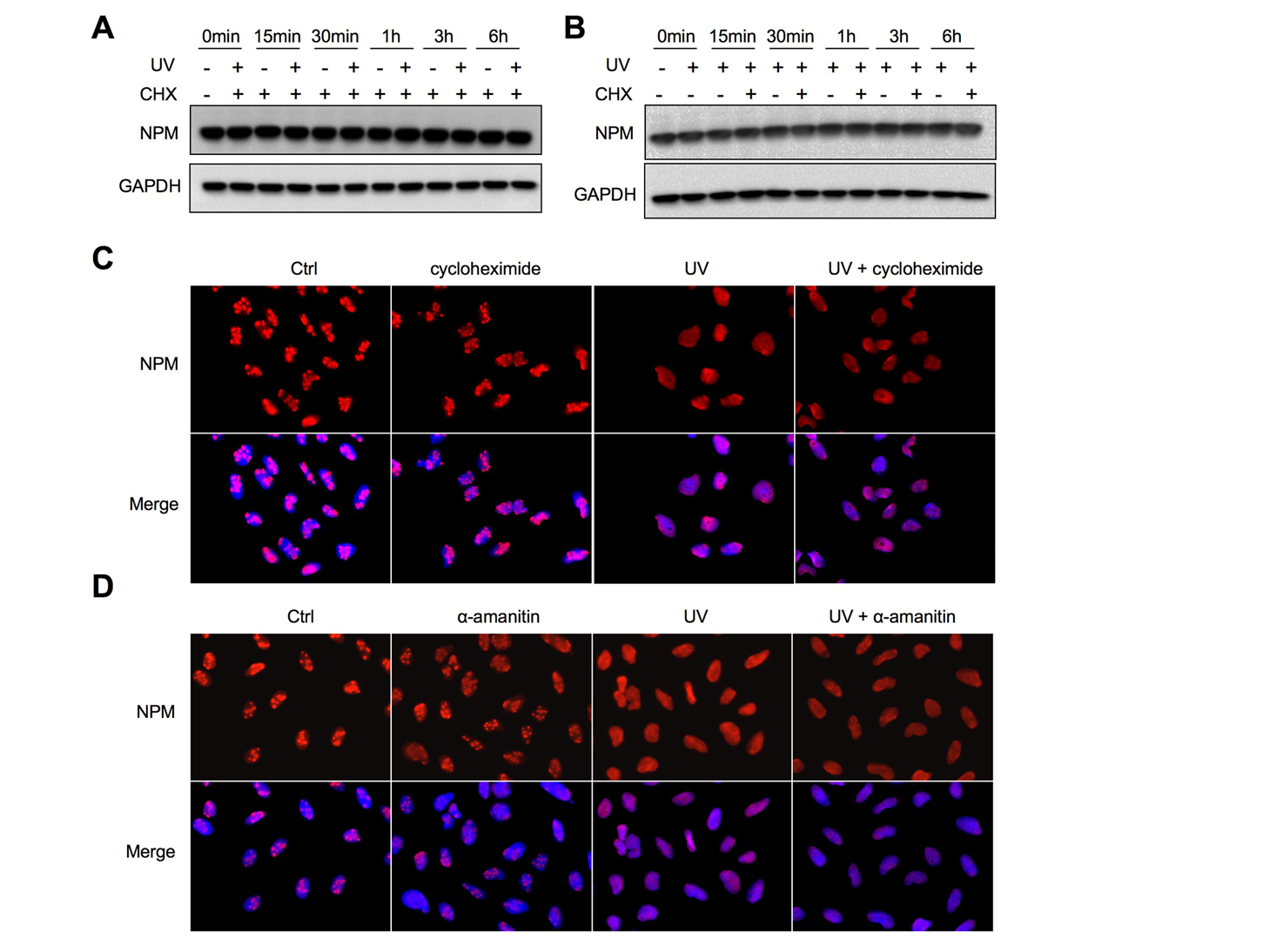

Supplement: Figure S4 — NPM half-life is unaltered following UV damage. A and B, U2OS cells were treated with UV (35 J/m2) and incubated in the presence or absence of cycloheximide (CHX, 50 µg/ml) for the indicated times. Cell lysates were prepared and analyzed by immunoblotting for NPM and GAPDH as control. C, U2OS cells were treated with UV (35 J/m2) in the presence or absence of cycloheximide (50 µg/ml) and incubated for 3 h. Fixed cells were stained for NPM (red) and DNA (blue). D, U2OS cells were treated with UV (35 J/m2) in the presence or absence of α-amanitin (25 µg/ml) and incubated for 3 h. Fixed cells were stained for NPM (red) and DNA (blue). (TIF) [file pone.0059096.s004.tif]

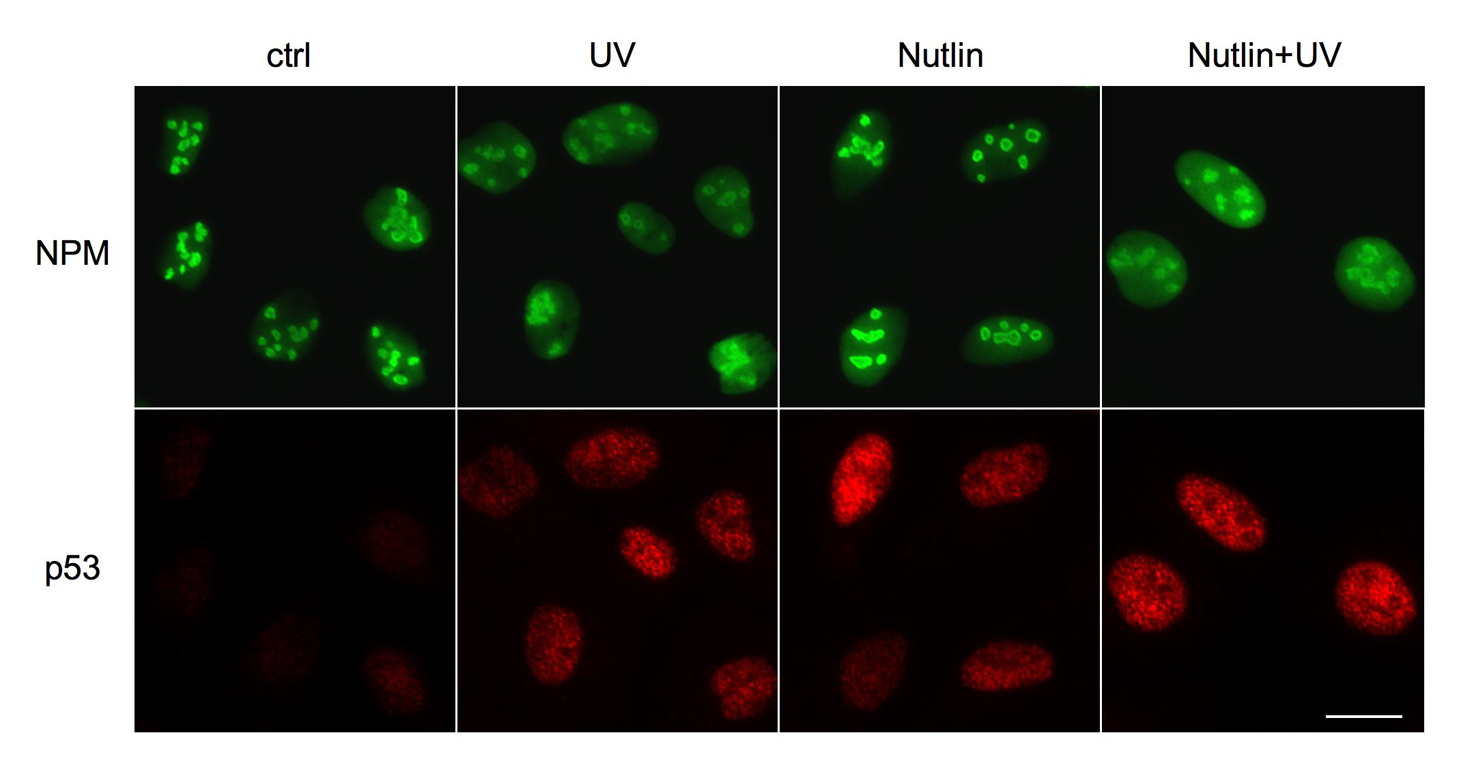

Supplement: Figure S5 — Nutlin-3 does not affect NPM redistribution after UV. U2OS cells were treated with either Nutlin-3 (10 µM) or UV (35 J/m2), or pretreated with Nutlin-3 for 1 hour followed by UV treatment and incubated for 3 hours, or left untreated (control). The cells were fixed and stained for NPM and p53. Scale bar, 20 µm. (TIF) [file pone.0059096.s005.tif]

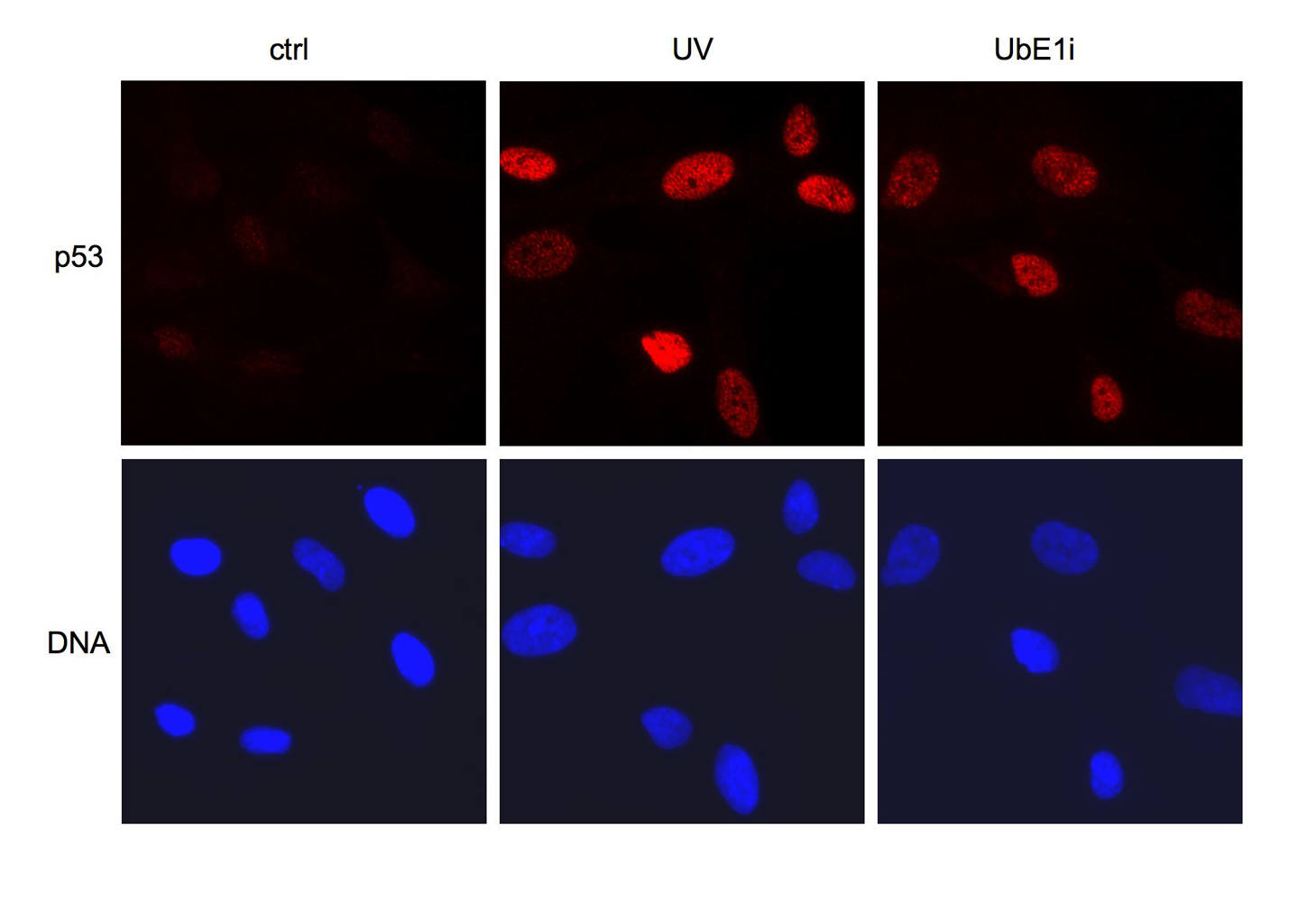

Supplement: Figure S6 — UbE1 inhibitor induces p53 response. WS1 cells were treated with UV (35 J/m2) or UbE1 inhibitor (10 µM) and incubated for 19 hours or left untreated. The cells were fixed and stained for p53. (TIF) [file pone.0059096.s006.tif]

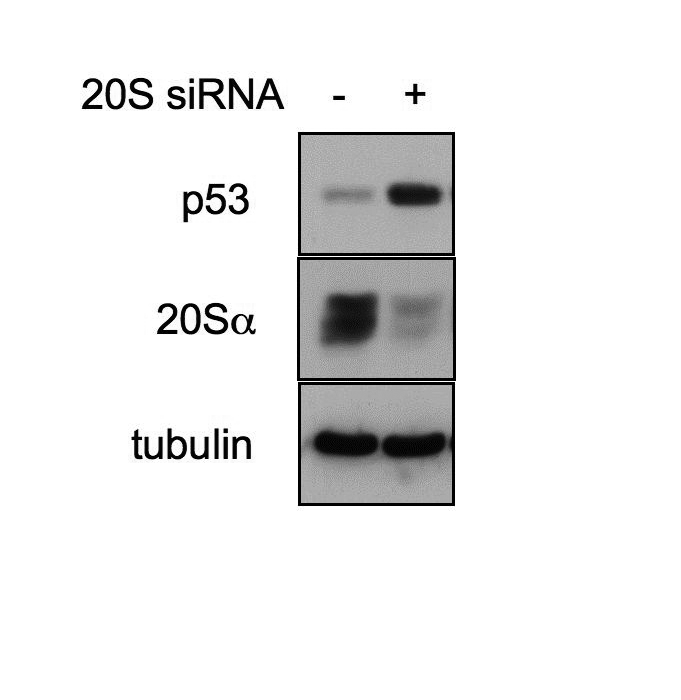

Supplement: Figure S7 — Silencing of 20S proteasome. HeLa cells were transfected with specific siRNAs against 20S α proteasome and the cells were incubated for 72 hours. Equal amounts of total protein were separated by SDS-PAGE and immunoblotted for p53 and 20S. Tubulin was used as a loading control. (TIF) [file pone.0059096.s007.tif]
